# Cervical Stenosis After Hysteroscopic Surgery for Cesarean Scar Disorder

**DOI:** 10.7759/cureus.56922

**Published:** 2024-03-25

**Authors:** Naofumi Higuchi, Yusuke Sako, Kyoko Shiota, Tetsuya Hirata

**Affiliations:** 1 Department of Obstetrics and Gynecology, St. Luke’s International Hospital, Tokyo, JPN; 2 Department of Obstetrics and Gynecology, St. Luke's International Hospital, Tokyo, JPN

**Keywords:** cervical stenosis, complication, hysteroscopic surgery, cesarean scar defect, cesarean scar disorder

## Abstract

Cesarean scar disorder (CSDi) is a newly recognized cause of secondary infertility. Laparoscopic or hysteroscopic surgery is generally chosen for the surgical treatment of CSDi, depending on the residual myometrial thickness of the cesarean scar. Previously, hysteroscopic transcervical resection for CSDi (TCR-CSDi) has been reported to be a safe procedure, with no cases of postoperative cervical stenosis. Herein, we report a novel case of cervical stenosis after circumferential hysteroscopic TCR-CSDi of an extensive CSDi lesion. Notably, although no cervical stenosis was observed upon postoperative hysteroscopy one month postoperatively, cervical stenosis developed four months after the surgery; therefore, it is important to avoid circumferential resection and cauterization in patients with CSDi, even when abnormal blood vessels are present. Additionally, it is advisable to check for delayed cervical stenosis at least three weeks before embryo transfer in patients who have undergone TCR-CSDi.

## Introduction

Cesarean scar disorder (CSDi) is a newly recognized disease caused by a cesarean section that has presented various symptoms in recent decades [1]. Its frequency is estimated to be approximately 18-24% in cesarean section patients [1], and it is known by various names such as niche, isthmocele, cesarean scar syndrome, and cesarean scar defect [2]. Recently, a CSDi study group defined CSDi as a cesarean scar defect more than 2 mm deep with one or more primary symptoms or two or more secondary symptoms [2].

CSDi can cause abnormal uterine bleeding, chronic pelvic pain, and secondary infertility in women of reproductive age [2-4]. Although the cause of CSDi is not yet elucidated, there are reports describing fibrosis and adenomyosis as the main histopathologic features of cesarean scar defects [1]. In women who do not intend to become pregnant, treatment options for CSDi include medications such as oral contraceptives or the levonorgestrel-releasing intrauterine system; however, surgery is the only treatment for CSDi patients with infertility [1]. Due to the risk of uterine perforation at thinning scars when performing transcervical resection (TCR) for CSDi, laparoscopic or hysteroscopic surgery is generally chosen for treatment, depending on the residual myometrial thickness (RMT) [5].

TCR-CSDi has been reported to increase RMT and reduce symptoms of CSDi [1]. Previously, three methods of lesion resection for TCR-CSDi have been reported, including (i) resection of only the inferior edge of the defect, (ii) resection of the superior and inferior edges of the defect, and (iii) resection of the bottom of the defect in addition to the superior and inferior edges [1]. However, the best resection method has not yet been established. TCR-CSDi has a low complication rate and is considered a safe procedure [5]. Moreover, there have been no previous reports of postoperative cervical stenosis after TCR-CSDi.In this study, we aim to report a rare and novel case of cervical stenosis after TCR-CSDi.

## Case presentation

**Figure 1:**
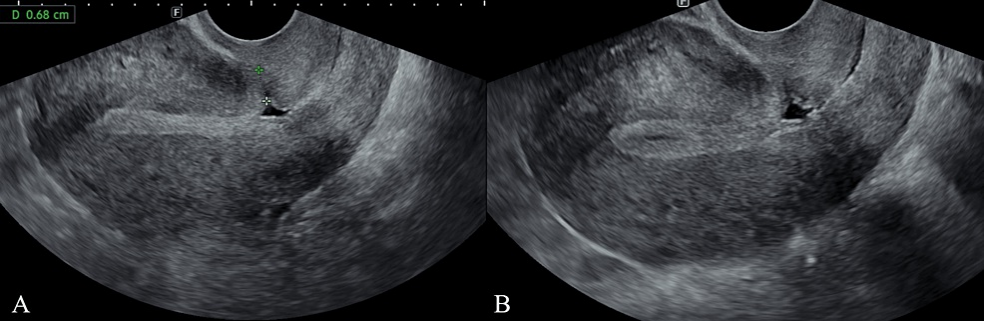
Transvaginal ultrasound images Transvaginal ultrasonography shows a cesarean scar defect with an RMT of 6.8 mm (A), as well as fluid retention in the uterine cavity (B). RMT: residual myometrial thickness

A 38-year-old gravida 3, para 1 woman presented to our hospital with a chief complaint of infertility. The patient had a history of an emergency cesarean section, indicating a non-reassuring fetal status. Hormonal tests, a chlamydia PCR test, and a semen analysis of the husband were performed to screen for infertility, all of which were normal. Fertility treatment was initiated one year after the cesarean section; however, no pregnancy was achieved for one year. The patient experienced persistent brown genital bleeding during this period, and transvaginal ultrasonography revealed a cesarean scar defect (RMT, 6.8 mm) and fluid retention in the lumen (Figure 1).

**Figure 2:**
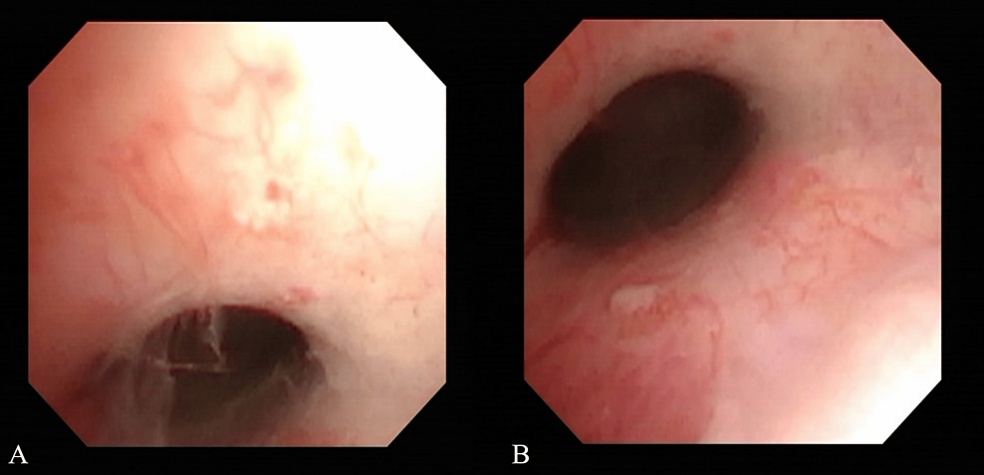
Preoperative hysteroscopic images Hysteroscopy reveals the presence of abnormal vessels in the scar (A) and in the circumference of the uterine isthmus (B).

Hysteroscopy revealed abnormal dendritic vessels in the scar, with a circumferential distribution (Figure 2).

**Figure 3:**
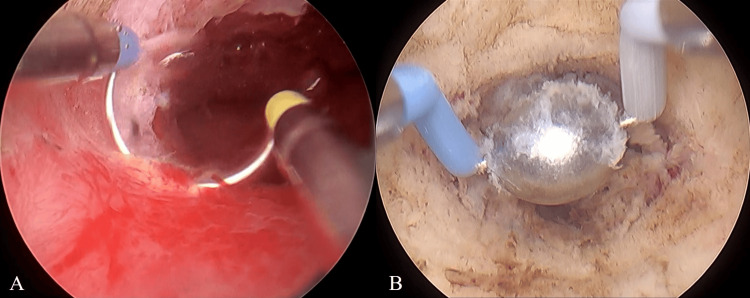
Operative findings during TCR-CSDi During TCR-CSDi, the uterine isthmus was resected (A) and coagulated (B) circumferentially, as well as in the scar. TCR-CSDi: hysteroscopic transcervical resection for cesarean scar disorder

Based on these findings, the patient was diagnosed with CSDi, and TCR-CSDi was planned. During surgery, in addition to the bottom and both the inferior and superior edges of the scar, the uterine isthmus was resected circumferentially with a loop electrode and coagulated with a ball electrode to cauterize the residual lesion (Figure 3). We utilize VIO 3 (Erbe Elektromedizin, Tubingen, Germany) as an electrosurgery generator with the setting of Effect 1 in high cut mode and Effect 4 in soft coag mode. The surgery was completed without any complications, with an operative time of 17 minutes and an estimated blood loss of 0 ml.

A second-look hysteroscopy performed one month postoperatively showed no adhesions, although the scar area was rough due to cutting and coagulation. Four months postoperatively, a frozen embryo transfer was performed under endometrial conditioning with hormone replacement. Under transabdominal ultrasound guidance, we attempted to insert a 3-Fr embryo-transfer catheter (1.9-mm outer diameter) after the bladder was sufficiently filled with saline solution. However, the catheter did not pass through the anatomical internal cervical os and showed strong resistance to insertion. Therefore, postoperative cervical stenosis, especially the narrowing of the uterine isthmus, was suspected. Under the guidance of transabdominal ultrasound tomography, the cervical stenosis was bluntly perforated using a uterine sonde, the catheter was successfully inserted into the uterine cavity, and the embryo was successfully transferred.

However, after the embryo transfer, the catheter was removed with strong resistance. At six weeks of gestation, the fetal heartbeat was confirmed; however, at seven weeks of gestation, the fetal heartbeat could no longer be confirmed, and the patient was diagnosed with a missed abortion.

## Discussion

Herein, we report a case of cervical stenosis diagnosed during embryo transfer after TCR-CSDi, in which we thoroughly resected the abnormal vessels around the uterine isthmus. Notably, although second-look hysteroscopy showed no cervical stenosis one month after surgery, cervical stenosis developed four months postoperatively. To the best of our knowledge, this is the first report of a case of cervical stenosis that developed after TCR-CSDi.

Cervical stenosis is a cause of secondary infertility [6] and an anatomical obstacle to artificial insemination and embryo transfer [7]; however, a clear definition for cervical stenosis has not yet been established [6]. Some reports define cervical stenosis as the cervix not allowing the passage of a 2.5-mm Hegar dilator [8], while others define this as an external cervical os diameter of <4.5 mm [9]. Additionally, a recent review by Vitale et al. defined cervical stenosis as a condition requiring a procedure to reach the uterine cavity [6]. According to this definition, our patient had cervical stenosis because the 1.9-mm embryo-transfer catheter failed to reach the uterine cavity during frozen embryo transfer, requiring subsequent blunt perforation with a uterine sonde under transabdominal ultrasound guidance.

Our patient developed cervical stenosis after TCR for CSDi. Conventionally, when treating CSDi, hysteroscopic surgery is performed when the patient has a sufficient RMT, whereas laparoscopic surgery is performed when the RMT is insufficient [1]. Although there have been no previous reports of cervical stenosis after hysteroscopic or laparoscopic surgery for CSDi, in this case, cervical stenosis may have been caused by the surgical method used for TCR-CSDi. Of the three methods of lesion resection used for TCR-CSDi, our institution typically performs resection of the inferior edge of the defect. However, in this case, due to the presence of abnormal blood vessels around the uterine isthmus, the upper, lower, and bottom parts of the defect, as well as the entire circumference of the uterine isthmus, were resected and coagulated using a method similar to the "channel-like (360°) hysteroscopic technique" reported by Casadio et al. [10]. This method has been reported to be more effective than the conventional method in improving the clearance of inflammatory substances and menstrual blood from the scar and promoting re-epithelialization of the uterine isthmus [11]. However, the risk of developing postoperative cervical stenosis is high with this method because cauterization of the endometrium, including the uterine isthmus, is a risk factor for cervical stenosis [12]. Another study previously reported that loop-electrode incision of the cervical lesion is another risk factor for cervical stenosis [8], suggesting a potential risk of cervical stenosis after TCR-CSDi. Furthermore, a report on cervical stenosis after conization stated that the incidence of postoperative cervical stenosis was significantly higher in cases in which >2 cm of the cervix was resected than in those in which <2 cm of the cervix was resected [8]. Thus, a larger resection area is more likely to result in cervical stenosis. Therefore, the occurrence of cervical stenosis in our case might partially have been due to the larger circumferential resection and cauterization in this method than in the conventional method. This shows that even if abnormal blood vessels are present, wide resection and especially circumferential ablation should be avoided when performing TCR-CSDi.

In the present case, the embryo transfer was difficult due to cervical stenosis, even though a second-look hysteroscopy one month postoperatively revealed no cervical stenosis. Most second-look hysteroscopies are performed approximately one to two months after hysteroscopic surgery [13-14]. However, delayed cervical stenosis was identified four months postoperatively in our patient. We hypothesize that large areas of resection and coagulation require more time for scar tissue to grow. We also speculate that the perfusion fluid in the second-look hysteroscopy may have inhibited adhesion formation, resulting in a delay in the completion of the cervical stenosis. Given the current case, in high-risk CSDi cases requiring extensive resection and cauterization, it may be advisable to check for delayed cervical stenosis by inserting a Hegar dilator into the cervix at least three weeks before embryo transfer [7].

## Conclusions

In patients with CSDi and secondary infertility, thorough hysteroscopic resection and cauterization of circumferential abnormal vessel lesions in the uterine isthmus may result in postoperative cervical stenosis. Therefore, even if circumferential abnormal vessels are present, it may be better to limit lesion resection to the bottom and the superior and inferior edges of the defect. Alternatively, using a Hegar dilator to check for delayed cervical stenosis before embryo transfer may be advisable for high-risk patients.
